# Acknowledgment to Reviewers of *Journal of Imaging* in 2020

**DOI:** 10.3390/jimaging7020014

**Published:** 2021-01-25

**Authors:** Journal of Imaging Editorial Office

**Affiliations:** MDPI AG, St. Alban-Anlage 66, 4052 Basel, Switzerland

Peer review is the driving force of journal development, and reviewers are gatekeepers who ensure that *Journal of Imaging* maintains its standards for the high quality of its published papers. Thanks to the cooperation of our reviewers, in 2020, the median time to first decision was 20.5 days and the median time to publication was 50 days. The editors would like to express their sincere gratitude to the following reviewers for their precious time and dedication, regardless of whether the papers were finally published:
Abdellatif, MohamedLin, XiaoAbdel-Nasser, MohamedLitany, OrAbel, AndrewLiu, BinAbelha Mota, Guilherme LucioLiu, Sheng-ChiAbir, MuhammadLiu, ShenghengAghaei, MayaLiu, SuxingAhmed, WaelLivieris, Ioannis E.Aldea, EmanuelLópez Granado, Otoniel MarioAlidoost, FatemehLukinac, JasminaAliotta, FrancescoLukomski, MichalAlmeida, MarcosLukoševičius, SauliusAl-qershi, OsamahLukosi, EricAlzubaidi, LaithLuqman, Muhammad MuzzamilAmbroziak, AndrzejMabrouk, RostomAmini, MarziehMachikhin, AlexanderAmiri, SamMagalhães, RicardoAmpeliotis, DimitrisMakek, MihaelAnagnostopoulos, IoannisMandal, SubhamoyAndreić, ŽeljkoMarasović, TeaAntunes, VanessaMarcato Júnior, JoséArguelles-Cruz, Amadeo J.Markelin, LauriAwad, Ali SaidMarone, FedericaAzetsu, TadahiroMartin, IainBacanin, NebojsaMartínez Torres, JavierBae, Sung-HoMartins, DianaBaek, SeungryulMascarenhas, Nelson D. A.Bahmanyar, RezaMasiero, AndreaBalado Frías, JesúsMastrotheodoros, Georgios P.Bamatraf, Saeed M.Matic, TomislavBarjaktarović, Marko Č.Matsubara, Edson TakashiBarker, Michael KennethMazurek, PrzemysławBarnes, AnnaMcKenna, Stephen J.Batchuluun, GanbayarMeerwald-Stadler, PeterBecek, KazimierzMeiburger, Kristen M.Beecks, ChristianMercier, GregoireBegušić, DinkoMerrill, FrankBelaid, AbdelMielewczik, MichaelBengtsson, EwertMigallón, HéctorBerg, Carl FredrikMihelič, JurijBernstein, Alexander V.Milicevic, MarioBevacqua, Martina TeresaMiller, AlexanderBevitt, JosephMiller, BorisBianconi, FrancescoMilosavljević, AleksandarBłaszczak-Bąk, WioletaMinaee, ShervinBobkowska, KatarzynaMishra, DeependraBobulski, JanuszMocanu, IrinaBobyr, MaximMoccia, SaraBock, Joel R.Moldovanu, SimonaBogunović, HrvojeMonno, YusukeBollmann, SteffenMontel, DeniseBordoy, JoanMontisci, AugustoBouhrara, MustaphaMoon, HyeonjoonBowman, AdrianMorgado, António MiguelBrehar, RalucaMorgano, ManuelBrudfors, MikaelMorillas Gómez, SamuelBuonamici, FrancescoMorris, ChristopherCalvo-Zaragoza, JorgeMousas, ChristosCândido Carvalho, KátiaMuhammad Arslan, UsmanCanetta, ElisabettaMüller, UlrichCarbon, Claus-ChristianMunaiz, Eduardo D.Caron, GuillaumeMuñoz, AdolfoCastejón-Limas, ManuelMyers, Kenneth A.Castellanos Regalado, Francisco JoséNagai, TakeharuCebollada, SergioNaik, Ganesh R.Celona, LuigiNakagawa, MasakiCernadas, EvaNavarro, NicolasChan, Kwok-PingNguyen, Van PhucChang, Kuo-JenNicholson, Kerry CawseChauhan, Vedang DilipkumarNicolas, StéphaneChaves, DeisyNocerino, EricaChen, Hung-ShingNorton, Kerri-AnnChen, SimingNuutinen, MikkoChen, Yu-JenO’Loughlin, DeclanChetouani, AladineO’Regan, DeclanChicchi Giglioli, Irene AliceOikawa, KenichiChoi, YonghoOkarma, KrzysztofChromy, AdamOlaru, MihaelaChuang, Tzu-YiOniga, FlorinChuirazzi, WilliamPaek, Sung WookCintra, Renato J.Paeng, Dong-GukCiuonzo, DomenicoPagnozzi, AlexCizelj, LeonPakula, AnnaCoccia, MarioPanchapakesan, BalajiCocianu, Cătălina-LuciaPandey, Prem ChandraConceicao, RaquelPaquet, ThierryCoronel, AnibalPark, Ji HwanCorti, MartinaParkinson, DilworthCosma, GeorginaParnell, StevenCraft, Aaron EPasquini, CeciliaCruz-Hernández, CésarPastena, MassimilianoCuocolo, RenatoPastorino, MatteoCzajkowska, JoannaPaternain, DanielDa Silva Soares, AndersonPelc, MariuszDalitz, ChristophPelt, Daniël M.Dang-Nguyen, Duc-TienPeng, Yan-TsungDaoud, BilelPeng, YifanDawson, JeremyPepe, Francesco V.De Marco, MatteoPeter, PascalDe Mingo López, Luis FernandoPetrellis, NikosDe Paiva, Anselmo CardosoPibernik, JesenkaDe Reijke, Theo M.Pino, Esteban J.Delibasis, Konstantinos K.Pires, Ivan Miguel SerranoDemidenko, EugenePiva, AlessandroDemidova, LiliyaPlech, AntonDeng, TiantaiPosada, JorgeDeniziak, StanisławPouli, TaniaDi, ZichaoPouliakis, AbrahamDiaz-Cortes, MargaritaPrasad, Dilip K.Diem, MarkusPuppe, FrankDiez, YagoPutzu, LorenzoDijkstra, JoukeQi, GuanqiuDingliana, JohnQi, ZhengDiraco, GiovanniQian, ChunqiDobrez, LivioRabaev, IrinaDogaru, RaduRadvan, RoxanaDomínguez-Morales, Juan P.Rahman, MahmudurDorizzi, BernadetteRao, Kamisetty R.Dorota, Majorkowska-MechRao, MuzaffarDoutch, JamesReber, JonasDoutsi, EffrosyniReeves, AdamDresp-Langley, BirgittaRefice, AlbertoEl Adoui, MohammedRen, WuweiEl Yacoubi, Mounim A.Ren, YuzhuoElbahnasawy, MagdyRengarajan, AmirtharajanElmogy, MohammedRenson, LudovicElwahab, Sami AbdReynolds, HayleyEstomba, Carlos Miguel ChiesaRibas, SalvadorFang, JianRichter, RudolfFanti, AlessandroRiebe, DanielleFarmer, MichaelRighi, MarcoFeng, Hsuan-MingRodriguez, Jeffrey J.Fernandes, Henrique C.Roger, Jean-ClaudeFernández-Caballero, AntonioRoig, Ignacio BoschFerrari, ClaudioRomanuke, VadimFerreira, ArturRomeo, StefaniaFerreira, FernandoRoșca, DanielaFesta, GiuliaRoy, SudiptaFeuerhake, FriedrichRuijsink, BramFinkbeiner, StevenRusu, MirabelaFiorentino, Maria ChiaraRuszczak, BogdanFlusser, JanRyu, KanghyunFonseca-Pinto, RuiSaabni, RaidForzani, EricaSafonov, IliaFox, RichardSaha, SudipanFreitas, Pedro GarciaSajjanhar, AtulFujiwara, TakeshiSalido Tercero, JesúsGade, RikkeSanchez, VictorGaffar, AshrafSanli, FusunGallego, Antonio-JavierSantos, FlorentinoGao, ZhanSawall, StefanGasparini, FrancescaSaxena, ShefaliGatto, Bernardo B.Schaff, FlorianGavrilov, DmitrySchillinger, BurkhardGavrilova, MarinaSchläffer, MartinGavrovska, AnaSchoenhagen, PaulGawronek, PelagiaSchregle, RolandGebremariam, Kidane FantaSebastião, HelderGerard, SarahSelci, StefanoGhidoni, StefanoSemeniuta, OleksandrGhosal, SambuddhaSequeira, AnaGiannakeas, NikolaosSerra-Sagristà, JoanGibbs, JohnSfarra, StefanoGiovanacci, DavidSgalaberna, DavideGolnabi, Amir H.Shafiq, OmairGrosse, MircoShah, JainilGuaragnella, CataldoSharma, AshutoshGuevara-Lopez, Miguel AngelSharma, SonisaGuidotti, PatrickShevkunov, IgorGünther, ManuelSiemiatkowska, BarbaraHakoyama, TomoyukiSimon-Chane, CamilleHamel, LutzSingh, SaurabhHarada, MasahideSkopal, TomášHarizanov, StanislavSmith, RhodriHautière, NicolasSobieranski, Antonio CarlosHavig, PaulSochen, NirHavlicek, Joseph P.Solano-Correa, Yady TatianaHeidari, MortezaSoltaninejad, MohammadrezaHellbourg, GregorySong, YoungseokHo, Edmond S. L.Sparavigna, Amelia CarolinaHorgan, GrahamSrinivasan, RaviHossain, BelayatStavropoulou, EleniHosseini, Seyed M. H.St-Charles, Pierre-LucHosu, VladStoean, CatalinHotz, IngridStojanovic, BrankaHożyń, StanisławStommel, MartinHsu, Chih-ChungStrobl, MarkusHuang, ShaoguangStrohmaier, Walter L.Huo, YumeiStrzelecki, MichalHussain, ZahirSu, HangHwang, Yin-TsungSu, YuhuaHwang, YoungbaeSujit, Sheeba JeniferIbarra-Manzano, OscarSulavko, AlexeyImani, MahdiSun, KeIngold, RolfSuwanwiwat, HemmaphanIntzes, IoannisSzczepanowska, HannaInzerillo, LauraTallents, Greg J.Ionescu, CringuTang, ZhaohuiIpek, ÖzlemTangherloni, AndreaIrianto, JeromeTan-Showyin, JackieIrrgang, ChristopherTarasov, AndreiIvashchenko, OlenaTarasov, DmitryJafri, RabiaTavakolian, PanteaJansing, DavidTeixeira, JoseJantzen, McNeel G.Teng, Liong SzeJaworska, TatianaTheodorakopoulos, IliasJeon, ByunghwanThomsen, KnudJester, JamesTian, Gui YunJochmanova, IvanaTinelli, AndreaJuan, GuillermoTinevez, Jean-YvesJubery, Talukder Z.Tonacci, AlessandroKaestner, AndersTrăscău, MihaiKamaleswaran, RishikesanTreimer, WolfgangKampa, AdrianTremsin, Anton S.Karakatič, SašoTrtik, PavelKardjilov, NikolayTrusiak, MaciejKarimov, TimurTzilas, VassiliosKatkovnik, VladimirUddin, JiaKhademi, AprilUlacha, GrzegorzKhan, Rizwan AhmedUllah, MohibKichanov, SergeyUllah, ZahidKim, WonjunValsesia, DiegoKim, Yoon-ChulValtchinov, Vladimir I.Kirchgessner, NorbertVan Eijck, LambertKlemeš, Jiří JaromírVasquez, Jorge A. TobonKline, Timothy L.Vassilakis, EmmanuelKnyaz, VladimirVaughan, Christopher L.Kockelmann, WinfriedVenkataraman, RajeshKoike, TakahikoVijayaraghavan, Gopal R.Kojima, TetsuyaVocaturo, EugenioKolb, AndreasWang, HaifengKopczyński, ErykWang, QianKorecki, PawelWang, ShuihuaKose, UtkuWang, ShuoKothapalli, Sri-Rajasekhar (Raj)Wang, ShuohongKoutsoudis, AnestisWatkins, EricKramer, TobiasWatson, DesKranjčić, NikolaWerman, MichaelKrejcar, OndrejWick, ChristophKrivoshein, A.Widerska-Chadaj, Zaneta SKumar, ShitijWilliams, JeremiahKuriki, IchiroWollmann, PhilippKwon, SunghoonWrzesiński, GrzegorzLadevèze, SandrineWysocki, MarianLagares, AlfonsoXiao, RanLaManna, JacobXu, XiaowenLanteri, LucaXu, YapingLara-Cueva, Román AlcidesYang, JieLavoué, GuillaumeYang, Lee-XiengLazzaretti, André EugênioYao, AipingLe, Hoang NganYasrab, RobailLèbre, Marie AngeYoo, Jung SunLee, HyunkwangYoon, HyungchulLee, JinseokYoussof Zadeh, VahabLee, KwonmooYu, Ting-ToLee, Sang JunYuan, YixuanLee, SanghoonZambrano-Martinez, Jorge LuisLee, SeunggyuZamfir, Rares Halbac-CotoaraLehmann, EberhardZeng, KaiLeishman, Robert C.Zhang, LeiLi, HaoliangZhang, MingLi, HongliangZhao, MingjunLi, Yung-HuiZhou, JunLin, Cheng-HungZolliker, PeterLin, Huei-YungZubrzycki, JarosławLin, Shinfeng


